# Unraveling Mechanisms of Enzymatic Browning in Nuts and Their Relationship with Pre- and Post-Harvest Factors: Management Strategies for Mitigation

**DOI:** 10.3390/molecules30193866

**Published:** 2025-09-24

**Authors:** Gabriela Gavilán-CuiCui, Ricardo Lagos-Muñoz, Felix Miguel Ellena, Antonio Di Matteo, Filis Morina, Cristian Meriño-Gergichevich

**Affiliations:** 1 Doctoral Program in Natural Resources Sciences, Universidad de La Frontera, Temuco 4811230, Chile; g.gavilan02@ufromail.cl; 2 Laboratory of Physiology and Plant Nutrition for Fruit Trees, Faculty of Agricultural Sciences and Environment, Universidad de La Frontera, Temuco 4811230, Chile; r.lagos07@ufromail.cl (R.L.-M.); felix.ellena@ufrontera.cl (F.M.E.); 3 Laboratory of Soil Fertility, Faculty of Agricultural Sciences and Environment, Universidad de La Frontera, Temuco 4811230, Chile; 4 Department of Agricultural Sciences, University of Naples Federico II, Via Università 100, 80055 Naples, Italy; antonio.dimatteo@unina.it; 5 Laboratory of Plant Biophysics & Biochemistry, Institute of Plant Molecular Biology, Biology Centre of the Czech Academy of Sciences in Budweis, 370 05 Ceske Budejovice, Czech Republic; morina@umbr.cas.cz; 6 Scientific and Technological Bioresources Nucleus (BIOREN-UFRO), Universidad de La Frontera, Temuco 4811230, Chile; 7 Department of Agricultural Production, Faculty of Agricultural Sciences and Environment, Universidad de La Frontera, Temuco 4811230, Chile

**Keywords:** enzymatic browning, polyphenol oxidase, peroxidase, phenolic compounds, physiological alteration, tree nuts, walnut green husk extracts

## Abstract

Enzymatic browning (EB) is a physiological alteration that compromises the sensory and commercial quality of tree nuts, significantly reducing their market value and functional compound content. Due to its complexity and economic impact, this review compiles updated information on mechanisms and factors driving EB in tree nut species, as well as strategies for its prevention. The EB in tree nuts results from the oxidation of phenolic compounds (PCs) to brown pigments. This process is driven by enzymatic activity such as polyphenol oxidase (PPO), peroxidase (POD), and phenylalanine ammonium lyase (PAL) and strongly enhanced by cellular stress and associated regulation of gene expression. The EB has been documented in several tree nut species, including almonds, betel nuts, chestnuts, hazelnuts, macadamias, pecans, pistachios, and walnuts. This alteration developed both pre-harvest and post-harvest and was influenced by agronomic factors (such as cultivar, nutritional status, climatic conditions, and altitude) and handling (including shelling, storage, and processing). Mitigation strategies include the use of synthetic inhibitors, physical treatments, and the use of plant extracts rich in natural antioxidants, the latter perceived as more sustainable and safer alternatives.

## 1. Introduction

Tree nut produce is of high commercial value at the global level. During the 2023/2024 season, their production reached 5.3 million metric tons (t), with an annual commercial value of USD $38 billion. In the last decade, world nut production has experienced an increase of nearly 50%, reflecting the growing demand and expansion of these products internationally. The United States leads global output with 50% of the total, followed by Turkey (14%), China (12%), Iran (5%), India (5%), Ivory Coast (4%), Australia (3%), Spain (2%), Vietnam (2%), and Chile (2%) [1].

The appearance, physicochemical properties, and nutritional value of nuts depend largely on the concentration and composition of their quality components, including proteins, vitamins, minerals, monounsaturated fatty acids (MUFAs), polyunsaturated fatty acids (PUFAs), and phenolic compounds (PCs) with high antioxidant properties [2,3]. Because of their rich nutritional content, nuts are highly valued by consumers, who demand rigorous standards for flavor, texture, and appearance [4]. However, the quality of tree nuts can be deteriorated significantly due to physiological alterations during the pre- and post-harvest stages [5].

One of the main physiological alterations that compromise the commercial and sensory quality of nuts is enzymatic browning (EB). This phenomenon is characterized by the appearance of brownish colorations resulting from enzymatic reactions leading to the loss of bioactive compounds, degradation of essential nutrients, and a reduction in shelf life [4,6]. The EB has been reported in several nut species, including almond (*Prunus dulcis* L.), betel nut (*Betel catechu* L.), chestnut (*Castanea* spp.), hazelnut (*Corylus avellana* L.), macadamia (*Macadamia* spp.), pecan (*Carya illinoinensis* L.), pistachio (*Pistacia vera* L.), and walnut (*Juglans regia* L.), representing a growing challenge for producers, processors, and exporters [7,8,9].

The EB is initiated by the disruption of cell membranes, caused by the accumulation of reactive oxygen species (ROS). This allows the interaction of oxidative enzymes with their substrates. In particular, polyphenol oxidase (PPO; EC 1.10.3.1) and peroxidase (POD; EC 1.11.1.7) catalyze the oxidation of phenolic compounds (PCs) to orthoquinones, which subsequently polymerize into melanins responsible for brown coloration [6,10]. In addition, phenylalanine ammonium lyase (PAL; EC 4.3.1.24), a key enzyme in the phenylpropanoid pathway, is involved in the biosynthesis of phenolic precursors that are also involved in browning [7,11]. This process negatively impacts nut acceptability in the market and can reduce the production by up to 30% [4,12,13]. For example, in 2007, EB caused losses of approximately USD $2 million to the macadamia industry in Australia, impacting both crop yield and processing efficiency [14].

The EB can manifest either while the nut is still on the tree or immediately after harvest. Its development at pre-harvest can be driven by agronomic and environmental factors including cultivar [15], mineral nutrition [16], climatic conditions, and altitude [17]. Post-harvest factors encompass shelling practices [5], storage conditions, and industrial processing parameters [18].

Three main strategies have been evaluated to prevent or mitigate EB in tree nuts. The first one consists of applying synthetic inhibitors, classified according to their mechanism of action as reductive [19], competitive [20], chelating [21], and complexing inhibitors [22]. The second strategy includes the application of physical treatments, including thermal methods [18], non-thermal technologies [23], modifications in the composition of the storage atmosphere [9], and the application of low temperatures [24]. Finally, the use of plant extracts has gained relevance as a natural alternative due to the presence of PC and antioxidants that inhibit enzymatic activity [25].

Owing to the complex nature of EB and its detrimental effects on commercial and sensory attributes of tree nuts, this review critically evaluates underlying biochemical mechanisms, pre-/post-harvest factors, and emerging mitigation strategies. In this context, prevention technologies are proposed that have the potential to strengthen research and practice, consolidate existing knowledge, identify key information gaps, and promote specific, sustainable, and applicable solutions throughout the supply chain.

## 2. Morphological Characterization of the Enzymatic Browning of Fruits in Tree Nuts

The EB in tree nuts generates significant morphological alterations, including progressive discoloration of tissues predominantly towards brownish tones, structural collapse of cell membranes, loss of turgor, as well as the appearance of cracks and fissures in different structures of the nuts. These changes are closely related to the increased activity of oxidative enzymes such as PPO, POD, and PAL, together with PC oxidation, processes favored by extrinsic factors that compromise the sensory, nutritional, and commercial quality of the product [8,20,22,25].

**Figure 1:**
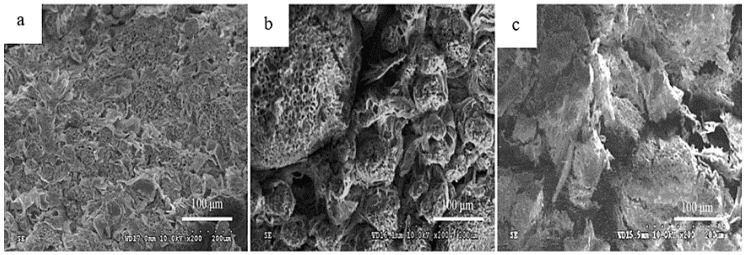
Scanning electron microscopy (SEM) of betel nut. In the initial stage of storage (0 days), the nut cross section is relatively flat and uniform, the tissue arrangement is neat and orderly, and the kernel structure is relatively complete, with no interstitial space or signs of EB (**a**). Toward the middle of storage (10 days), the arrangement of kernel tissues becomes disordered and the bonds between them begin to weaken (**b**). At the end of storage (20 days), the interstitial space of the kernel tissue increases, accompanied by structural damage and full development of EB (**c**) [27].

For example, in almond, EB is manifested by internal brown discoloration of the kernel, related to PAL and POD activity, in addition to high PC content, which generates bitter flavors that cause immediate consumer rejection [26] (Table 1). Similarly, in betel nut, prolonged storage causes cell membrane shrinkage, leading to cracks (Figure 1) and facilitating the entry of O_2_, increasing browning and EB development in the kernel [27] (Table 1). This same mechanism, based on cell membrane damage and adverse environmental conditions, such as high temperatures, humidity, and poor air circulation, explains EB in macadamia, where enzymatic activity, especially of PPO, increases PC oxidation, causing unpleasant odors and flavors and important economic losses [14,28,29] (Table 1).

**Figure 2:**
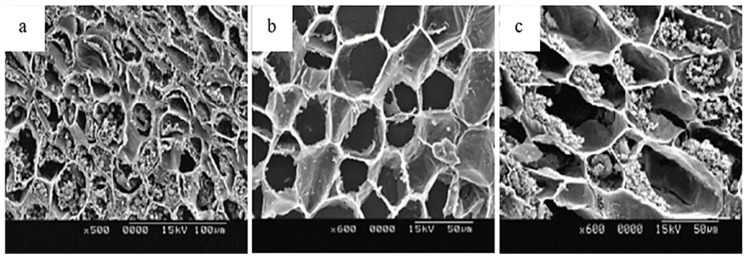
Scanning electron microscopy (SEM) representative of hazelnut with different degrees of browning. Affected cells show progressive loss of their cellular contents as a result of internal plasmolysis (**a**) until only the cell wall persists, which in later stages begins to gradually degrade (**b**), causing the collapse and rupture of the affected tissue, finally forming small holes that give rise to the internal cavity (**c**) [12].

In chestnut, hazelnut, pecan, and pistachio, EB affects both the shell and kernel and is strongly associated with PPO, POD, and PC activity. In chestnut, browning is intensified after peeling, mechanical damage, and prolonged exposure, accompanied by flavor alterations and nutritional losses, which negatively impacts consumer acceptance [6,7,21,22,30]. In hazelnut, agroclimatic factors influence EB, such as the main production zones, accumulation of heat units, and relative humidity during the final stages of kernel development (mid-July) [17]. It starts with a brown liquid at the apex and progresses from the softened shell towards the interior of the kernel, generating stains and moisture (Figure 2), compromising its integrity and commercial value, since affected kernels tend to fragment more easily during industrial processing [12,17,31] (Table 1). In pecan, EB occurs as a gradual color change in the kernel testa, from golden yellow to brownish, due to high PPO activity (up to 1442 times higher than initial values) and PC content during storage, which affects flavor and nutritional quality [8,32] (Table 1). In pistachio, the hull is especially vulnerable to post-harvest browning and during storage, associated with the progressive increase of PPO and POD, which catalyze the oxidation of PC, favoring the development of EB and notably decreasing quality [10,19,20] (Table 1).

**Figure 3:**
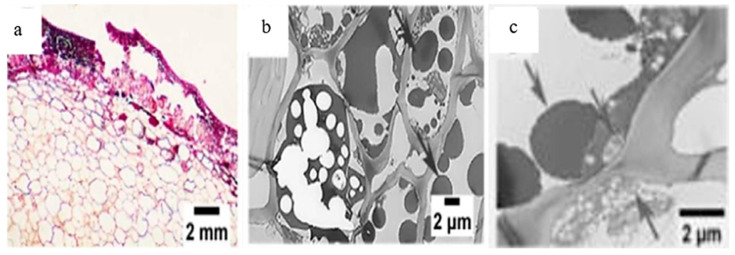
Ultrastructural images of the epicarp and its cell walls in the hull. A damaged epicarp with marked darkening of the nut was observed (**a**). At the cellular level, numerous black substances were identified in vacuoles, mitochondria, and chloroplasts (**b**,**c**) (black arrows) [9].

Finally, in walnuts, EB affects both the kernel and the hull. Kernel browning is linked to storage conditions and shelling, processes that favor PPO-mediated enzymatic oxidation of PC, reducing quality and antioxidant capacity. For its part, the hull shows microstructural alterations and the appearance of a black substance (Figure 3) that impedes its protective function against oxidation, which decreases the commercial value of the nut [5,9,25,33,34] (Table 1).

**Table 1:** Description of enzymatic browning symptoms observed in commercial tree nuts.

Tree Nuts	Description of Symptoms	Image	References
Almond (*P. dulcis* L.)	It covers 50% of the inner part of the kernel with a brown coloration, associated with bitter flavors, which can develop both during and after harvest.		[26]
Betel nut (*B.catechu* L.)	The kernel gradually decomposes with darker color changes; it is destroyed in the form of a honeycomb, decreasing lignification and increasing the interstitial space.	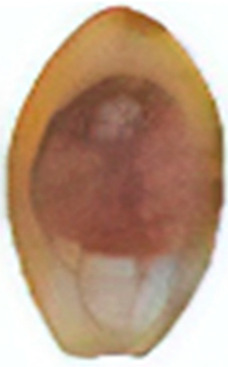 ^1^	[27]
Chestnut (*C. mollissima Blume*) (*C. henryi*)	Kernels turn brown after processing, changing color and causing degradation of flavor and nutrients, thus reducing shelf life.	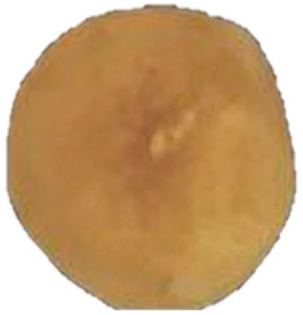 ^2^	[6,7,21]
Halzenut (*C. avellana* L.)	The cells in the browning zone gradually lose their isodiametric shape until the tissue is destroyed by lysis which results in the appearance of brown watery exudates and generates a softer and less consistent nut.	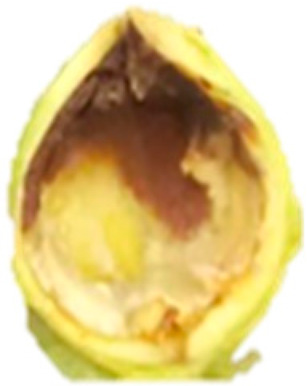 ^3^	[12,31]
Macadamia (*M. integrifolia*) *(M. tetraphylla)*	A thin line of brown discoloration around the equator of the kernel, which in severe cases affects the entire base of the kernel, accompanied by an unpleasant odor and taste.		[14,28,29]
Pecan (*C. illinoinensis* L.)	Freshly harvested kernels have a golden yellow color, which gradually turns brown during storage, affecting flavor, nutritional value, and commercial appeal.	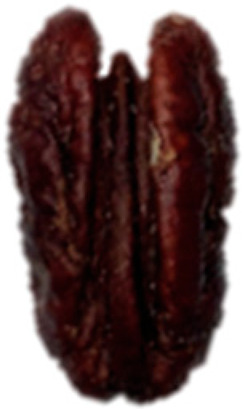 ^4^	[8,32]
Pistachio (*P. vera* L.)	It is present in the hulls, causing a brown coloration followed by putrefaction, causing quality loss during processing and storage.		[10,20]
Walnut (*J. regia* L.)	The kernels and hull are prone to darken, forming a black exudate that contaminates the inside of the nut, thus reducing quality.	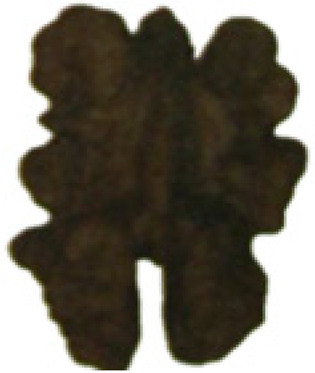 ^5^	[4,5,9,33]

The images were extracted from the following publications: ^1^ [27]; ^2^ [7]; ^3^ [31]; ^4^ [32]; ^5^ [5].

## 3. Mechanisms of EB of Commercial Tree Nuts and Their Relationship with the Different Systems Involved

**Figure 4:**
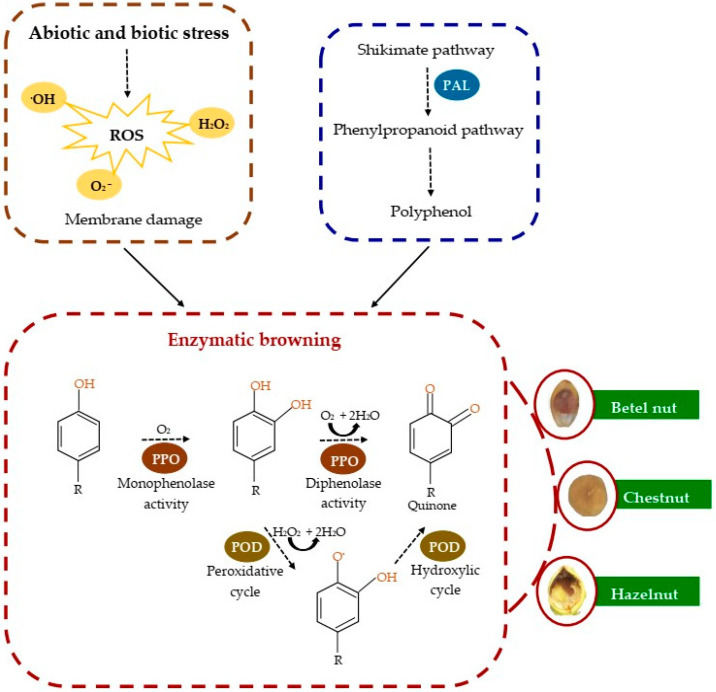
General outline of the EB mechanism in tree nuts, illustrating the main biochemical stages and enzymatic interactions involved in the oxidation of phenolic compounds, the formation of quinones, and the consequent generation of brown pigments responsible for sensory and commercial deterioration.

As shown in Figure 4, ROS such as superoxide (O_2_^−^), hydrogen peroxide (H_2_O_2_), and hydroxyl radical (•OH) are natural products of cellular metabolism, and their concentration is regulated by an antioxidative system (enzymatic and non-enzymatic antioxidants). However, excessive ROS accumulation due to abiotic or biotic stress causes oxidative damage to lipids, proteins, and DNA, compromising the cellular integrity and inducing browning, spoilage, and quality deterioration in nuts [4,9]. As a consequence of cellular damage, the loss of subcellular compartmentalization allows the contact between PPO and PCs to initiate EB. The PPO is an oxidoreductase that contains copper in its structure and uses O_2_ as an electron acceptor to oxidize PCs [25,35]. This enzyme catalyzes two fundamental reactions: the oxidation of monophenol to orthoquinone (monophenolase activity) and the oxidation of diphenol to orthobenzoquinones (diphenolase activity), which directly leads to tissue browning [19,27]. Another key enzyme in this process is POD, which contains a heme group in its structure and is also involved in PC oxidation, contributing significantly to browning [36]. The POD acts through two distinct mechanisms: a peroxidative cycle that utilizes H_2_O_2_ to oxidize phenolics to phenoxy radicals and a hydroxylic cycle that reduces H_2_O_2_ to •OH [36]. The POD can use substrates other than those used by PPO, which contributes to the formation of phenolic radicals and quinones, acting in a complementary and synergistic manner in the EB reaction [36]. Phenolics, originating from the shikimate and phenylpropanoid pathways, as well as by the action of the PAL enzyme, have an aromatic ring with one or more hydroxyl groups, whose arrangement in the ortho position favors their oxidation by PPO and POD [7,11,22]. The result of this oxidation leads to the formation of quinones, highly reactive compounds that produce brown polymeric pigments and can polymerize through non-enzymatic reactions to form melanin, as well as react with amino acids, proteins, and phenols, ultimately causing browning [37,38]. This process results in a significant loss of nutritional and organoleptic qualities of the affected tissue, including a darker color, less firm textures, and the appearance of unpleasant odors [8,10].

In relation to the key factors triggering EB in tree nuts, as summarized in Table 2, the predominant involvement of several species-specific enzymes and PC oxidation has been identified. In betel nut, the main enzyme involved in EB is PPO, which catalyzes the oxidation of substrates such as chlorogenic acid, dopamine, and L-epicatechin [27]. In cashew, a correlation has been observed between PPO activity and the presence of compounds such as caffeic acid, catechin, gallic acid, and *o*-dihydroxyphenol catechol, identified as relevant substrates [23,37]. In chestnut, it was determined that the PAL enzyme participates in the generation of PC precursors of EB, such as catechol, chlorogenic acid, 2-hydroxy phenol, and *p*-hydroxybenzoic acid. In addition, the rapid increase in PPO and POD enzyme activity accelerates the oxidation of these PCs to quinones, which favors faster EB development [6,7,22,30]. In hazelnut, significant levels of PPO and POD activity were reported, which favored greater EB development. Coumaroylquinic and *p*-coumaric acids were also identified as low molecular weight monomeric substrates that were particularly efficient in the oxidation reactions catalyzed by these enzymes [31]. In macadamia, it was concluded that PPO activity was mainly responsible for EB, with chlorogenic and p-hydroxybenzoic acid being the main substrates involved [29]. In pecan, a direct relationship was determined between PPO activity and compounds such as catechin, 2,3,4-trihydroxybenzoic acid, phloroglucinol, procyanidin, and protocatechuic acid, all showing an upward trend in their involvement in EB [8]. In pistachio, PAL and PPO activities were observed to increase in parallel with the progression of EB, although PC oxidation was relatively low during this process [20]. In walnut, PPO was identified as the predominant enzyme in EB development, as various PCs such as caffeic acid, catechol, chlorogenic acid, coumaric acid, 4 hydroxyphenylacetic acid, epicatechin, ethyl gallate, gallic acid, guaiacol, L-DOPA, protocatechuic acid, and quercetin generated significant quantities of quinones, which self-polymerized to form brown pigments [13,39,40]. Finally, in Chinese walnut (*J. cathayensis* Dode), it was determined that chalcone synthase (CHS), 4-coumarate-CoA ligase (4CL), and PAL enzymes promoted an increase in PC. In addition, the active presence of PPO facilitated EB development [11].

On the other hand, recent studies on EB have highlighted a close relationship between gene expression, enzyme activity, and PC metabolism, which are key factors in the development of browning in commercial tree nuts. In almonds, Sarami et al. [26] determined that the peroxidase (*per67*) and chorismate mutase 2 (*CM2*) genes are associated with the activity of PAL and POD enzymes, which play a key role in the EB process. In Chinese walnut, Zhang et al. [11] and Persic et al. [41] identified two *PAL* genes, one *CHS* gene, and two *4CL* genes that showed significant differential expression during EB, promoting the corresponding enzyme activity and favoring an increase in PCs derived from the phenylpropanoid pathway. In pecan, Yang et al. [8] carried out a transcriptomic analysis by sequencing, identifying two essential genes related to PPO synthesis: *CiPPO1* and *CiPPO2*. These genes directly regulate the EB process, mainly through the PPO synthesis and subsequent accumulation of brown pigments associated with the melanin. In studies by Wang et al. [13], Panis and Rompel [39], and Zhao et al. [40], they concluded that the *JrPPO1* and *JrPPO2* genes play a key role in EB, as they contribute to the encoding and increased activity of PPO, generating the oxidation of phenols and the production of a large amount of quinones.

## 4. Factors Contributing to EB and Quality Deterioration in Commercial Tree Nuts

### 4.1. Pre-Harvest Factors

#### 4.1.1. Cultivar

The incidence of EB seems to be related to the genetic component, as it prevails over external factors. In fact, hazelnut cultivars such as Barcelona, Tonda Gentile delle Langhe, and Negret are considered the most sensitive to EB, with symptoms appearing between 154 and 176 days after fruit set (October–November, southern hemisphere), when the kernel reaches a height of 14.5 to 17.9 mm, coinciding with the final phenological phase of growth, affecting up to 97% of the total crop [12,17,31]. In macadamia, several cultivars were tested, and most showed a similar level of susceptibility to EB, attributable to the enzymatic activity of PPO [29]. However, the Daddow cultivar showed the lowest phenolic concentration (500 mg gallic acid equivalent kg^−1^ DW) compared to the others (A38, 246, 816, and 842), which was associated with lower susceptibility to EB-affected kernel formation, as phenolic substrate limitation resulted in minimal enzyme activity [29]. In walnut, eight cultivars were evaluated with respect to the development of EB, with different rates of speed in the manifestation of the disorder: rapid (Xifu2, Shaanhe, and Weina), intermediate (Xiangling), and delayed (Xiluo3, Xifu1, Liaoning4 and Qingxiang) development. Correlation analysis revealed that EB indices were significantly associated with PPO activity, LOX, and malondialdehyde levels, suggesting a link between these biochemical parameters and the susceptibility of cultivars to browning [15].

#### 4.1.2. Plant Nutrition

Several studies highlighted the effects of deficiency in certain mineral elements on development and incidence of EB in tree nuts. Deficiencies in calcium (Ca), boron (B), and zinc (Zn) are particularly implicated, as they are critical for nut set, reduced bunch drop, prevention of blank nuts, yield enhancement, quality improvement, and strengthened plant nutrition [42]. In addition, these elements mitigate oxidative stress by reducing ROS accumulation mainly through cell membranes [42]. In this context, Cacka and Smith [43] reported that foliar applications of Ca, B, and other micronutrients during hazelnut embryo/kernel development (mid-June) improved kernel/glomerule ratio by 22%, kernel weight by 9%, kernel/shell ratio also by 9%, and reduced EB incidence by 56%. First during shell development (mid-May) and second. Results indicated that applications made in June and spring applications of calcium nitrate (CaNO_3_) to the soil also improved hazelnut quality with a 49% increase in kernel yield, a reduction of mold by 37–41%, and a decrease in EB level by 44% [16]. The authors concluded that EB is a symptom of Ca deficiency, since the accelerated growth enlarges the central cavity of the nut, causing collapse and necrosis of the inner lining.

Contrastingly, nitrogen (N) excess could increase the incidence of EB by promoting nut deformations that compromise tissue integrity. However, Romero et al. [17] reported that N and B applications during late nut development stages explained only 2.5 and 4% of the total variation in EB incidence, respectively, proving negligible impact despite confirmed B uptake.

#### 4.1.3. Climatic Conditions and Altitude

Changes in climatic conditions, especially extreme fluctuations in temperature and rainfall or sudden hail, can affect the quality of tree nuts and increase the susceptibility to EB; in fact, these climatic conditions significantly influence the occurrence of EB in hazelnuts grown in Spain, as described by Romero et al. [17]. The main associated factor is low accumulation of heat units (AHU, r = −0.80) during the period from 110 to 131 days after nut set, which corresponds to the final stage of hazelnut kernel growth (mid-July), accompanied by mean relative humidity (MRH, r = 0.80), which has a crossover effect, increasing the development of EB. Therefore, these results suggest that climatic factors such as temperature and MRH are closely related to this physiological disorder, since the risk of EB increases when AHU is low and MRH is not high and decreases when AHU is high, especially if accompanied by a high MRH [17].

Another relevant factor is altitude, since an increase in the incidence of EB has been observed between 6 and 16% for every 100 m increase in altitude. These data coincide with the reports of Romero et al. [17], who recorded an incidence of EB in hazelnut trees of between 41 and 97% at an altitude of 500 m. Moreover, high temperature stress during nut development can cause reduced kernel weight and quality and increase kernel browning [44].

### 4.2. Post-Harvest Factors

#### 4.2.1. Nut Shelling

Shelling is one of the main operations of post-harvest processing of nuts, during which the outer skin is separated from the shell by applying compressive, manual, or mechanical forces that damage the kernel. Shelling has been suggested to be one of the factors contributing to the development of EB, due to tissue breakdown that induces such deterioration in nuts [5,36]. In a study by Ortiz et al. [5], they evaluated the effects of soft (<4% of the damaged film area) and hard (20–22% of the damaged area) shelling on Howard and Chandler walnut cultivars. Results showed that shelling intensity significantly affected kernel film integrity, leading to greater tissue browning, lipid degradation, and antioxidant loss in Howard kernels than in Chandler kernels. On the other hand, Christopoulos and Tsantili [36] investigated the post-harvest behavior of shelled and unshelled walnuts stored at low temperatures (1–8 °C), where the authors deduced that shelling slightly increased kernel browning at the lower temperature, while kernels that remained in the shell presented a lower incidence of the disorder. This was attributed to the protective effect of the shell, which limits O_2_ exposure and reduces the risk of physical damage during shelling.

#### 4.2.2. Storage and Industrial Processing

The EB develops under specific conditions of storage and processing of tree nuts, which negatively impact their quality. One of the main factors associated with its occurrence is the presence of moisture in the nuts. Le Lagadec [14] indicated that storage of macadamia with high moisture content favors the formation of EB in the center of the nuts, turning them brown. Likewise, Walton et al. [28] concluded that keeping macadamias with moisture levels above 20% increased the incidence of EB in 44% of the nuts. Therefore, it is recommended to reduce moisture content to 7–10% in the orchard and ship nuts in a timely manner to processing centers [14]. In addition to moisture, other post-harvest conditions that favor EB development include high temperatures during drying. In fact, Christopoulos and Tsantili [33] demonstrated that exposure to temperatures of 20 °C increased browning, decreased antioxidant content, and accelerated the deterioration of nut quality. In turn, Li et al. [18] reported that increasing the drying temperature to 80 °C in walnuts caused an increase in the intensity of browning, associated with the alteration of 64 heat-sensitive metabolites, demonstrating a strong impact of heat treatment on walnut integrity.

## 5. Strategies to Prevent Browning in Tree Nuts

Different strategies have been proposed to limit EB, such as the use of synthetic inhibitors, physical treatments, and plant extracts, which are detailed below.

### 5.1. Synthetic Inhibitors by Mode of Action

#### 5.1.1. Reduction

Synthetic reducers modulate enzymatic activity through their ability to reduce o-quinones, reverting them to their original phenolic substrate forms [19,35]. This group of inhibitors includes ascorbic acid (AA), sulfur dioxide, and other compounds containing thiol groups (such as L-cysteine and glutathione). According to Najjari and Bodaghi [19], a 6% concentration of AA was the most effective in reducing browning (19%), weight loss (3%), electrolytes (22%), and peroxide content (23%) in pistachio. Also, AA effectively preserved total phenolic compounds (TPCs) and inhibited PPO activity, which contributed to EB reduction and quality preservation (Table 3). In turn, Habibie et al. [35] assessed the effects of antioxidant edible coatings combining walnut green husk extracts (WGHE; 0.15 and 0.3 g L^−1^) and AA (1%) on kernel post-harvest quality in walnut. The authors reported an increase in TP levels, browning inhibition, reduction of peroxide indices and PPO activity, in addition to preservation of antioxidant capacity (AC) and sensory properties (Table 3).

#### 5.1.2. Competitive

They are characterized by reversible binding of enzyme molecules involved in EB, mainly through hydrogen bonds and hydrophobic interaction, which hinders the enzymatic reaction by competing with the substrate for the active site [6,20,45]. A member of this category is salicylic acid (SA), a plant hormone essential in the regulation of physiological processes that have great potential to reduce post-harvest loss [45]. Thus, Peng and Jiang [45] found that in fresh-cut Chinese water chestnuts (CWCs), treatment with SA delayed discoloration, maintained edible quality, and reduced PPO, POD, and PAL activities (Table 3). Similarly, Zhou et al. [6] reported that immersion SA treatment at concentrations higher than 0.3 g L^−1^ delayed browning in CWC by competitively inhibiting PPO activity; however, POD activity was not affected (Table 3). Another example of the competitive mechanism is the combination of edible coatings based on polysaccharides (alginate) and essential oils, used to prolong shelf life by reducing moisture and solute migration, gas exchange, respiration, and oxidative reaction rates, as well as to suppress physiological disorders [20]. In particular, coating pistachio-coated with 1% sodium alginate plus 0.3% thyme essential oil maintained quality for approximately 39 days of storage, while it decreased PPO activity and increased TP and PAL activity through hydrophobic interaction (Table 3). Conversely, uncoated pistachios showed more intense shell browning [20].

#### 5.1.3. Chelation

These compounds are characterized by their ability to remove metal ions present in the active sites of enzymes, causing their inactivation. The complexes they form with pro-oxidant metals, such as copper in PPO and iron in POD, act as EB inhibitors [21]. The main chelating agents include ethylenediaminetetraacetic acid (EDTA), oxalic acid (OA), and phytic acid (PA). In this context, Li et al. [21] evaluated the inhibitory effect of PA on browning and the mechanisms associated with enzymatic activity in fresh-cut chestnuts (CCs). Soaking peeled and cut chestnuts in a 0.01% PA solution suppressed external and internal browning, reducing the specific activities of PPO and POD by 80% and 76%, respectively, due to the competitive inhibition exerted by PA. However, despite its nature as a chelating agent, this study determined that PA acts as a competitive inhibitor for both enzymes (Table 3).

#### 5.1.4. Complexation

Their hydrophobic nuclei can form complexes with various molecules, thus preventing their oxidation to quinones and the consequent formation of EB. An example of the aforementioned corresponds to eugenol (EUG), the main component of clove (*Syzygium aromaticum* L.) essential oil, which utilizes antioxidant phenolics to inhibit free radical polymerization [7,22]. In fact, treatment with 1.5% EUG maintained CWC quality and inhibited browning by reducing enzyme activities (PPO, POD, and PAL), as well as changes in phenolic content and quinone formation (Table 3). Correlation analysis between enzyme activities and degree of browning suggested that PAL was the main enzyme leading to EB, as its activity decreased by 57% with EUG [7]. Furthermore, treatment with 1.5% EUG inactivated EB-related enzymes and reduced the phenolic content in the outer tissue of CWC [22]. Molecular docking confirmed the interactions and hydrogen bonds between EUG and PAL. In addition, EUG enhanced the ROS scavenging enzymatic activities, thereby decreasing the generation of O_2_. As for the internal tissue, EUG induced the accumulation of colorless PC and increased AC (Table 3). Another example of a complexing agent corresponds to nitric oxide (NO), a highly reactive free radical gas involved in stress response, senescence, and browning [10,30]. In fact, NO treatment (5 μm) delayed browning, inhibited enzymatic activities, and increased TPC; this effect was linked to the reaction of NO with copper and iron within the enzyme to form complexes that altered the active site structure (Table 3) [30]. Similarly, Gheysarbigi et al. [10] evaluated the inhibitory effect of sodium nitroprusside (SNP; NO donor) as an anti-browning agent in pistachios to control EB. Nuts treated with NO (15 and 30 μM) showed a lower browning index, reduced enzyme activity, and more stable TPC, flavonoid, and antioxidant activity, which prolonged post-harvest life (Table 3).

### 5.2. Physical Treatments

#### 5.2.1. Heat Treatment

Blanching is a thermal process, which involves the application of heat at a predetermined temperature for a specific time. It prevents undesirable enzymatic reactions, thus inhibiting EB during processing, contributing to the improvement of color, sensory qualities, and storage stability. Conventional hot water blanching is one of the most common processes due to its simplicity. However, it has some disadvantages, including high water and energy consumption, reduced enzymatic inactivation efficiency, and loss of water-soluble substances such as phenols [32]. In contrast, blanching with infrared radiation (IR) requires little water during the process and has proven to be effective in inactivating enzyme activity and enhancing product quality [32]. Specifically, IR blanching at 600 W significantly reduced the post-drying time in pecans and yielded nuts with lower peroxide values, browning index (BI), and quinone content [32]. This improvement stems from IR’s effective inactivation of lipoxidase (LOX, EC 1.13.11.) and PPO. In fact, IR-treated nuts showed residual activity levels of 16–40% for LOX and 17–56% for PPO, significantly lower than the 47–92% residual activity observed in hot water blanching. In addition, IR blanching resulted in higher TP retention (25–30 mg GAE g^−1^) indicating improved nut nutritional quality (Table 4). Another key thermal process is hot air drying, a widely used technique in the food industry. This process substantially delays EB by removing water, thereby inhibiting the chemical reactions triggering EB [18]. Li et al. [18] identified sixty-four metabolites related to EB in walnut pellicles. In particular, 14 flavonoids showed a strong correlation with BI, with a positive regulation of >60% upon EB. While most of the anthocyanins identified showed a negative relationship with BI due to degradation (>45%), with correlation coefficients ranging from 0.75 to 0.97. In addition, laccase (LAC: EC 1.10.3.2) and anthocyanidin reductase (ANR: EC 1.3.1.77) were identified as the two key enzymes involved in EB, presenting correlation coefficients with BI of 0.92 (LAC) and 0.77 (ANR). The increase in drying temperature during HD treatment resulted in enhancing LAC and ANR enzyme activities by 10.57- and 1.32-fold, respectively (Table 4).

#### 5.2.2. Alternative Treatment (Non-Thermal)

High hydrostatic pressure (HHP) is a non-thermal technology applied in the processing of agricultural products. It operates in a range of pressures between 100 and 1400 MPa, which does not alter the covalent bonds of the compounds relevant for nut quality. This property allows modulation of enzymatic activity, either by inactivation or activation, with the aim of controlling biochemical processes such as ripening, fermentation, or various specific enzymatic transformations, without compromising the molecular integrity of the heat-sensitive components and operating with uniformity and instantaneously [23]. Unlike thermal processes, HHP is independent of sample volume and shape, which reduces the time required to process large quantities of food. In this context, Queiroz et al. [23] reported that treatments applied to cashew nuts at 250 MPa and a temperature of 27 °C produced losses in color parameters, in addition to reducing the content of AA (14%), polyphenols (13%), and AC (41%), causing browning. The decrease in AA levels could favor PPO activity, since AA is a potent antioxidant known to inhibit the formation of browning compounds (Table 4).

#### 5.2.3. Atmosphere Composition Management

The controlled atmosphere (CA) storage refers to the control of gas composition around the fruit after harvest, generally by modifying the ratio of CO_2_ to O_2_. This reduces physiological activities such as respiration and ethylene release from nuts by regulating metabolic pathways, thereby prolonging shelf life, improving quality, and delaying EB [4,9]. The CA storage shares similarity with modified atmosphere (MAP), although the latter technology involves the extraction of air inside the package by introducing a specific gas mixture, adjusted to the type of product. This atmosphere is dynamically modified depending on biochemical changes and gas diffusion to the outside of the package. The beneficial effects of MAP include decreased respiratory activity, weight loss, protection against mechanical damage, delayed ripening, and minimized incidence of EB [34,46]. The MAP is classified into two types: passive and active. The passive method depends on the respiration rate of the product and the permeability of the pellicles used to produce its effects, while the active method creates an atmosphere that evolves according to storage conditions [47]. In this context, Ye et al. [4] investigated the effect of CA storage on quality parameters and the incidence of browning in walnuts. The results indicated that CA with a composition of 5% O_2_ and 7.5% CO_2_ promoted enzymatic activities, preserved TPC and ASA and reduced glutathione (GSH). Also, this condition inhibited ethylene synthesis, and reduced ROS as well as acid and peroxide values, which contributed to retarding kernel browning. Additionally, CA achieved higher kernel quality by inhibiting the decrease in color value and moisture content (Table 4). Similarly, Ye et al. [9] evaluated the regulatory effect of CA on the incidence of EB in-shell kernels, with the aim of elucidating the underlying physiological mechanisms. For this purpose, different cultivars were stored under CA conditions (5% O_2_ + 7.5% CO_2_) for a period of 70 days. The results showed that CA significantly increased γ-aminobutyric acid (GABA) levels, which was associated with increased glutamate decarboxylase, GABA transaminase, and succinate dehydrogenase activity. This reduced changes in BI, respiration rate, and alterations in color parameters in the four cultivars evaluated (Table 4). Consequently, the application of CA effectively controlled EB by enhancing GABA metabolism and preserving energy balance during storage.

**Table 4:** Physical treatments used to inhibit EB.

Treatment	Technique	Tree Nuts	Effect	References
Thermal	IR blanching	Walnut (*J. regia* L.)	It reduced drying time, peroxide values, browning, and quinone content, and inactivated LOX and PPO enzymatic activities. It also showed higher TP retention.	[32]
Thermal	Hot air drying	Walnut (*J. regia* L.)	Fourteen flavonoids were identified that showed a strong correlation with BI. In addition, LAC and ANR were the key enzymes involved in EB.	[18]
Non-thermal	High hydrostatic pressure (HHP)	Cashew (*A. occidentale* L.)	It produced losses of color parameters, in addition to the reduction of AA, polyphenols, and AC, causing EB.	[23]
Composition of the atmosphere	Controlled atmosphere (CA) storage	Walnut (*J. regia* L.)	It promoted enzymatic activity, preserved TPC, ASA, and GSH, inhibited ethylene production, and reduced ROS, acidity, and peroxide, delaying EB. It also increased quality by inhibiting color and moisture loss.	[4]
Composition of the atmosphere	Controlled atmosphere storage (CA)	Walnut (*J. regia* L.)	Increased GABA levels through increased activity of glutamate decarboxylase, GABA transaminase, and succinate dehydrogenase. Reducing changes in EB, respiratory rate, and color parameters.	[9]
Composition of the atmosphere	Modified atmosphere storage (MAP)	Almond (*P. dulcis* L.)	It showed no changes in film color, respiration intensity, or enzymatic activities of PPO and POD while fully maintaining their quality.	[46]
Composition of the atmosphere	Modified atmosphere storage (MAP)	Hazelnut (*C. avellana* L.)	Reduced lipid oxidation, BI, and quality deterioration.	[48]
Composition of the atmosphere	Modified atmosphere storage (MAP) active	Pistachio (*P. vera* L.)	It prevented weight loss and chlorophyll and carotenoid content. It also promoted higher levels of PAL while preserving levels of AC, TAA, TPC, and quality.	[47]
Composition of the atmosphere	Modified atmosphere storage (MAP)	Walnut (*J. regia* L.)	The levels of TP, total flavonoids, PAL, and antioxidant activity remained high, while the incidence of rot, PPO, and POD, in addition to peroxide value and acidity, was lower.	[34]
Low temperatures	Low temperatures	Walnut (*J. regia* L.)	Prevented loss of antioxidants and EB. In addition, increased TP and TAC in the stored walnut.	[33,36]
Low temperatures	Low temperatures	Walnut (*J. regia* L.)	It reduced oxidative metabolism, decreased PPO activity, suppressed antioxidant loss, and delayed EB.	[49]
Low temperatures	Low temperatures	Walnut (*J. regia* L.)	Decreased EB level, peroxide values, and PPO activity. As well as an increase in AC and TP levels, improving quality.	[24]

On the other hand, Massantini et al. [46] studied different types of almond storage under a modified atmosphere system (MAP) to preserve kernel quality. The results indicated that MAP with N_2_ at 4 °C was the most suitable to maintain chemical, physical, and sensory attributes, keeping their quality intact for almost two weeks of storage. However, almonds stored in air at +10 °C showed increased browning (Table 4). Moreover, Bai et al. [48] investigated the effects of MAP on quality deterioration of hazelnuts packed with 100% N_2_ and stored at 35 °C. As a result, MAP effectively reduced lipid oxidation, BI, and quality deterioration during 9 months of storage (Table 4). On the other hand, Sheikhi et al. [47] investigated the effects of passive and active MAP packaging on physicochemical characteristics of in-shell stored pistachio. The results showed that treatments with active MAP (2.5% O_2_ + 45% CO_2_) were more effective than passive MAP (21% O_2_ + 0.03% CO_2_ + 78% N_2_) in preventing weight loss and chlorophyll and carotenoid content, as well as maintaining AC and anthocyanin content. Active MAP treatments promoted higher levels of PAL enzyme activity (3.69 U mg^−1^ protein) than passive MAP while preserving TAA and TPC levels and post-harvest quality. Whereas, Wang et al. [34] investigated the effects of MAP packaging on phenol metabolism and in-shell walnut preservation, highlighting that high levels of TP, total flavonoids, PAL, and antioxidant activity were maintained. However, the incidence of rot, PPO, and POD activities, in addition to the peroxide value and acidity of oils under MAP conditions was lower upon storage (Table 4).

#### 5.2.4. Low Temperatures

Low temperatures slow down all chemical and metabolic processes by reducing the rate of oxidizing enzymes. Therefore, a decrease in temperature causes a delay in undesirable changes in nuts during storage, which will be greater the lower the temperature. As an example, Christopoulos and Tsantili [33] reported that low temperature (1 °C) prevented both antioxidant losses and EB in nuts. Likewise, Christopoulos and Tsantili [36] reported that TP and TAC increased 1.2- and 1.3-fold, respectively, in walnuts stored at 1 °C for 20 days (Table 4). Similarly, Shojaei et al. [49] reported that low temperatures (4 °C) slowed oxidative metabolism, decreasing enzymatic activities, in addition to suppressing antioxidant depletion, which delayed EB of walnuts for 6 months (Table 4). Similarly, Shojaee et al. [24] reported that samples stored at 4 °C presented the lowest level of EB, peroxide value, and PPO activity, as well as an increase in AC and TP, which significantly improved walnut quality (Table 4). On the other hand, samples stored at 25 °C presented the highest EB, accompanied by a higher peroxide value and lower amounts of antioxidants and TP.

### 5.3. Tree Nut Extracts as Potential Inhibitors of EB

In the last two decades, the demand for new natural agents that can replace chemicals used to limit EB has increased, as consumers prefer safer, environmentally sustainable methods, which at the same time preserve sensory properties [35]. Plant residue extracts are natural agents that are inexpensive and easily accessible raw materials containing PC with strong antioxidant activity capable of neutralizing free radicals to maintain cell membrane stability and mitigate oxidative enzymes that cause EB [35]. For example, WGHE, a by-product of walnut cultivation, is rich in antioxidants that help preserve their properties by inhibiting both lipid oxidation and EB [35,50]. In this regard, Chatrabnous et al. [50] evaluated the influence of WGHE on the inhibition of biochemical changes in walnut quality. The results revealed that WGHE inhibited lipid peroxidation, increased the acidity index, improved the quality, and prolonged the post-harvest life during storage due to the bioactivity of different PCs (Table 5). On the other hand, Habibi et al. [25] and Habibie et al. [35] evaluated the effects of WGHE in combination with AA (1%) on walnut quality during storage. Plant extracts maintained higher levels of AC and TP and inhibited H_2_O_2_ accumulation and PPO activity, thus preserving nut quality attributes (Table 5). Given the scarcity of available studies, it is essential to further investigate the potential of plant residue extracts as EB inhibitors in tree nuts in order to take advantage of their bioactive compounds and promote their valorization.

## 6. Conclusions and Perspectives

The EB is a physiological alteration of great relevance that affects numerous tree nut species. It is caused by the formation of brown pigments derived from the enzymatic oxidation of PC to quinones, which negatively affects their visual quality, nutritional value, commercial acceptance, and industrial yield. The EB development is closely related to pre-harvest and post-harvest factors that trigger biochemical responses mediated mainly by enzymes such as PPO, POD, and PAL, together with the participation of key regulatory genes such as *per67*, *CM2*, *PAL*, *CHS*, *4CL*, *CiPPO1*, *CiPPO2*, *JrPPO1*, and *JrPPO2*, directly involved in the molecular mechanisms of browning.

This review also addressed three main strategies to limit EB: the use of synthetic inhibitors that interfere with enzyme activity; physical treatments aimed at reducing the enzyme kinetics of the process; and the use of plant extracts rich in natural antioxidants, the latter being an alternative of growing interest due to their safe, sustainable profile and compatibility with current consumer requirements.

Finally, we emphasize the need for more comprehensive and multidisciplinary research that will allow a deeper understanding of the various mechanisms involved in the development of EB. It is also proposed to incorporate new lines of study that include the analysis of the role of the microbiota associated with tree nuts and its potential influence on oxidative metabolism; the study of the interaction between variable climatic conditions and the generation of oxidative stress; the evaluation of epigenetic responses induced by agricultural practices and post-harvest treatments; and the implementation of emerging technologies for the early detection and efficient control of EB.

## Figures and Tables

**Table 2:** Enzymes and substrates involved in the browning of tree nuts.

Tree Nuts	Enzymes	Phenolic Substrates	References
Betel nut (*B. catechu* L.)	PPO	Chlorogenic acid, dopamine and L-epicathechin	[27]
Cashew (*A. occidentale* L.)	PPO	caffeic acid, catechin, gallic acid and o-dihydroxyphenol catechol	[23,37]
Chestnut (*C. mollissima L.*)	PAL PPO POD	Catechol, chlorogenic acid, 2-hydroxy phenol and *p*-hydroxybenzoic acid	[7,30]
Hazelnut (*C. avellana* L.)	PPO POD	Coumaroylquinic and *p*-coumaric acid	[31]
Macadamia (*M. integrifolia* L.)	PPO	Chlorogenic acid, *p*-hydroxybenzoic acid	[29]
Pecan (*C. illinoinensis* L.)	PPO	Catechin, 2,3,4-trihydroxybenzoic acid, phloroglucinol, procyanidin, and protocatechuic acid	[8]
Pistachio (*P. vera* L.)	PAL PPO	-	[20]
Walnut (*J. regia* L.)	PPO	Caffeic acid, catechol, chlorogenic acid, coumaric acid, 4 hydroxyphenylacetic acid, epicatechin, ethyl gallate, gallic acid, guaiacol, L-DOPA, protocatechuic acid, and quercetin	[13,39,40]
Walnut (*J. cathayensis* Dode)	CHS 4CL PAL PPO	-	[11]

**Table 3:** Synthetic inhibitors used to inhibit EB.

Mechanism	Treatment	Tree Nuts	Effect	References
Reduction	Ascorbic acid (AA)	Pistachio (*P. vera* L.)	It reduced browning, weight loss, electrolytes, and peroxide. In addition, it preserved TPC and inhibited PPO activity, reducing EB.	[19]
Reduction	Ascorbic acid (AA)	Walnut (*J. regia* L.)	Inhibited EB, peroxide oxidation, and PPO. In addition to increasing TPC, AC preservation, and sensory properties.	[35]
Competitive	Salicylic acid (SA)	Chinese chestnut (*Eleocharis dulcis*)	Delayed discoloration, maintained quality, and reduced PPO, POD, and PAL activities.	[45]
Competitive	Salicylic acid (SA)	Chinese chestnut (*C. mollissima*)	It delayed EB by inhibiting PPO activity, while POD was unchanged.	[6]
Competitive	Sodium alginate + thyme oil	Pistachio (*P. vera* L.)	Quality was maintained, since PPO activity decreased, and TPC and PAL increased.	[20]
Competitive	Phytic acid (PA)	Chestnut (*C. mollissima blume*)	Due to the inhibition of external and internal EB, PPO, and POD activities also decreased.	[21]
Complexation	Eugenol (EUG)	Water chestnut (*Eleocharis* *tuberosa*)	Maintained quality and inhibited EB by reducing PPO, POD, and PAL activities, as well as changes in PC and quinone formation.	[7]
Complexation	Eugenol (EUG)	Water chestnut (*E. tuberosa*)	Inactivated EB-related enzymes and reduced the phenolic content of the external tissue. It increased ROS enzymatic activity, decreasing O_2_. In addition, PC accumulation occurred, and internal tissue AC increased.	[22]
Complexation	Nitric oxide (NO)	Chestnut (*C. henryi*)	It delayed EB and inhibited PPO and POD activities while TPC increased.	[30]
Complexation	Sodium nitroprusside (SNP)	Pistachio (*P. vera* L.)	It showed a lower browning index and reduced PPO, PAL, and POD activity. In addition to maintaining the levels of TPC, flavonoids, and AC, prolonging post-harvest life.	[10]

**Table 5:** Natural agents used to inhibit EB.

Treatment	Technique	Tree Nuts	Effect	References
Walnut green husk extract (WGHE)	Natural agent	Walnut (*J. regia* L.)	Inhibited lipid peroxidation, increased acidity index, improved quality, and prolonged post-harvest life.	[50]
Walnut green husk extract (WGHE)	Natural agent	Walnut (*J. regia* L.)	Increased AC and TP levels prevented the increase of peroxide value and PPO activity, inhibited EB, and preserved quality attributes.	[25,35]

## Data Availability

Data are contained within the article.
